# Control externo de la calidad en medicina del laboratorio. Avances y futuro

**DOI:** 10.1515/almed-2022-0059

**Published:** 2022-08-12

**Authors:** Carmen Ricós, Pilar Fernández-Calle, Carmen Perich, Sverre Sandberg

**Affiliations:** Sociedad Española de Medicina del Laboratorio (SEQC^ML^), Barcelona, España; Departamento de Medicina del Laboratorio, Hospital Universitario La Paz, Madrid, España; Organización noruega para la mejora de la calidad de los exámenes del laboratorio (NOKLUS), Hospital Universitario Haukeland, Bergen, Norway

**Keywords:** aseguramiento de la calidad, control externo, especificaciones de la prestación

## Abstract

**Objectivos:**

Un programa de control externo distribuye las mismas muestras control entre varios laboratorios y evalúa los resultados obtenidos con un criterio común. El objetivo de este trabajo es resumir la evolución de los programas externos, poner de manifiesto los progresos conseguidos y deducir consecuencias prácticas para el laboratorio participante.

**Métodos:**

El material es una breve revisión de los diferentes tipos de programas externos utilizados a lo largo de cuarenta años. El método es el análisis crítico de las ventajas e inconvenientes de cada modelo, a la luz de nuestra experiencia.

**Resultados:**

A mitad del siglo XX se iniciaron los programas EQA, detectándose gran discrepancia entre resultados emitidos por distintos laboratorios. Se desarrollaron EQA en muchos países y se propusieron mecanismos para armonizarlos, como: establecer especificaciones derivadas de la variación biológica, promover el uso de métodos analíticos homogéneos, usar el EQA como herramienta educacional. A partir del 2000 se hacen importantes avances: asegurar el adecuado uso clínico de las pruebas del laboratorio, utilizar material control conmutable con el espécimen humano, armonizar los distintos modelos de EQA, promover una organización de cooperación entre organizadores de programas EQA.

**Conclusiones:**

Participar en un EQA con controles conmutables y valores asignados por método de referencia certificado permite conocer la inexactitud real de los resultados y el impacto en las muestras de pacientes. Si se participa en programas con controles no conmutables solo se conoce si la prestación del laboratorio es similar a la de otros usuarios del mismo método analítico.

## Introducción

La medicina del laboratorio es una ciencia pionera en el desarrollo y aplicación del control externo de la calidad de sus procedimientos analíticos. Un programa de control externo consiste en un procedimiento de reparto de las mismas muestras control entre varios laboratorios y la evaluación de los resultados obtenidos, por parte de una organización externa al laboratorio. El término generalmente aceptado para esta actividad es Aseguramiento (o garantía) externo de la calidad (EQA por las siglas en inglés *de External Quality Assessment*) [1].

Otro término también usado es Prueba de Aptitud (*Proficiency Testing*), que es algo muy parecido al control externo de la calidad que ofrece un reconocimiento formal de la obtención de buenos resultados por parte del participante [2, 3].

Este estudio está enfocado exclusivamente al control externo de la calidad de la fase analítica del laboratorio médico y no se refiere a la fase extra-analítica.

El objetivo de este trabajo es resumir la evolución de los programas externos, mediante el ejemplo de los programas de la SEQC, poner de manifiesto los progresos conseguidos y deducir consecuencias prácticas para el laboratorio participante y para los organizadores de los mismos.

## Materiales y métodos

El material es una breve revisión de los diferentes tipos de programas externos que han sido utilizados a lo largo de cuarenta años.

El método es el análisis crítico de las ventajas e inconvenientes de cada modelo, a la luz de nuestra experiencia.

## Resultados

### Antecedentes históricos

El primer programa de aseguramiento Externo de la Calidad conocido data de 1947, fue introducido por Bellk y Sunderman en los Estados Unidos [4]; era una disolución acuosa de 9 constituyentes bioquímicos que se distribuyó a unos 60 laboratorios y puso de manifiesto que un 15% de los resultados se identificaron como errores importantes, alrededor de un 50% fueron insatisfactorios y un 35% se consideraron como satisfactorios.

El primer programa de hematología se creó en 1969 por Lewis y Burgess [5] y en los años 70 en la conferencia de Atlanta [6] e inmediatamente después la IFCC publicó su recomendación provisional *External* *Quality Assessment*, también denominado ejercicio de inter-comparación, que en 1983 se convirtió en recomendación aprobada [7]. En aquel momento, los objetivos fundamentales de los EQA fueron:

– Documentar la inexactitud de los resultados del laboratorio individual.
– Verificar la imprecisión para los programas en que el material control era analizado en replicados, en comparación con la obtenida por los restantes laboratorios participantes.
– Evaluar la aceptabilidad de los resultados con criterios estadísticos.

Brevemente, se creó también el control externo de las fases extra-analíticas, siendo el *College of American Pathologists* (CAP) la primera organización que desarrolló este tipo de programa [8, 9]. La SEQC ofrece varios programas de control externo para la fase extra-analítica [10–12].

La SEQC inició su experiencia en control externo en 1977, distribuyendo entre sus socios el programa de bioquímica de la *Société Française de Biologie Clinique*, consiguiendo una inscripción de 60 laboratorios con una participación activa del 50%.

En 1980 organizó su primer programa EQA para bioquímica en suero, que fue evaluado y acreditado por la Organización Mundial de la Salud (OMS) en 1984. Entre los años 1989 y 2002 desarrolló un programa pluridisciplinario (bioquímica, hematología y microbiología), mediante un acuerdo con la Asociación Española de Hematología y Hemoterapia y con un grupo de microbiólogos. En adelante, se desarrollaron varios nuevos programas (orina, hormonas (I y II) en suero, proteínas en suero, gases en sangre, glicohemoglobina en sangre, marcadores cardíacos en suero y marcadores tumorales en suero, fármacos en suero, drogas de abuso en orina).

El comité de programas externos de la SEQC se implicó en todo momento en el trabajo de grupos de expertos internacionales, con el objeto de mejorar continuamente sus programas y mantenerlos en la primera línea de conocimientos, dentro de las posibilidades en nuestro país.

Hasta ese momento el análisis de los resultados de la participación de los laboratorios era meramente descriptiva, mediante un estudio estadístico de la dispersión de los resultados de los participantes y la comparación de los resultados individuales frente a una media global o consenso y cada uno de los organizadores y programas tenía establecidos criterios propios de análisis y de cumplimiento.

En los años 90 el *Standards Measurement and Testing Programme*, desarrolló criterios para estandarizar los EQA existentes en aquellos momentos [13]:

– Definir las especificaciones de la calidad para los EQA.
– Promover el uso de métodos analíticos homogéneos (calibrador, reactivos, instrumento de un mismo fabricante).
– Seleccionar el mejor material control posible.
– Evaluar la prestación de cada laboratorio individual y de los métodos participantes con criterios similares entre los distintos EQA.
– Enfocar el EQA con un propósito educacional.

Se recomendó el uso de especificaciones de la calidad analítica basadas en la variación biológica tanto para el control interno [14] como para el externo; esto último se fundamentó en la evidencia de enormes discrepancias entre EQA como por ejemplo Colesterol donde se aceptaba una desviación respecto al valor diana del 3% hasta el 18% según el país organizador del EQA [15]. Se explicaron también los requisitos necesarios para la elección del material control en programas de suero [16]. Para evaluar la prestación de los laboratorios participantes, se recomendó establecer los valores diana a los materiales mediante su análisis por métodos de referencia [17] y se inició la creación de una red de laboratorios con estos métodos [18].

Todo este conocimiento fue reconocido en la norma internacional ISO-Guide 43 sobre Pruebas de aptitud mediante comparación entre laboratorios [19].

Ya en 1998 la Directiva Europea 98/79/EC [20] estableció que los EQA deben verificar la armonización entre laboratorios usuarios de los métodos analíticos disponibles en el mercado. Un programa EQA tiene por tanto como objetivo la comparación entre laboratorios diseñada y realizada para asegurar alguno de los siguientes aspectos: la evaluación de la prestación del participante, la evaluación de la prestación del método analítico, la vigilancia de los sistemas de diagnóstico *in vitro* y la educación continua, entrenamiento y ayuda a los participantes.

En 2002 la IFCC dictó una serie de recomendaciones a los organizadores de programas de control externo para acreditar su competencia técnica en su rol de vigilancia [21]. La IFCC en ese momento definió un nuevo tipo de EQA, que denominó: Programa de Garantía Externa de la Calidad (*External Quality Assurance Program, EQAP*), para diferenciarlo de los hasta entonces llamados programa de evaluación externa de la calidad (EQAS). La palabra “garantía” en este contexto se diferencia de “evaluación” en que no se limita a evaluar la prestación analítica, sino que incluye la interpretación de las pruebas de laboratorio y aconseja al clínico sobre la capacidad diagnóstica de las mismas. A esto se le añade el objetivo de formación y educación continuada de los profesionales del laboratorio. El objetivo de un EQAP en la medicina del laboratorio es, por tanto, impulsar la calidad del servicio prestado por el laboratorio, para el beneficio del paciente.

Aunque las siglas EQAP no se han usado mucho, la mayoría de los proveedores de programas EQA ofrecen también “garantía” de la calidad.

La organización EQALM (*European Organization for External Quality Assurance Providers in Laboratory Medicine*), usa el término “garantía” o “aseguramiento” para señalar lo que se practica realmente en la mayor parte de los programas EQA. EQALM es una organización que engloba a los organizadores europeos de EQA en medicina de laboratorio. EQALM provee un foro de cooperación e intercambio de conocimiento sobre temas relacionados con la calidad, con especial enfoque en los programas EQA existentes en Europa.

En esta revisión se utiliza el término original EQA de forma genérica, porque no es nuestro objetivo resaltar las diferencias entre las distintas siglas reconocidas.

### Avances de los EQA en el siglo XXI

En las primeras dos décadas del siglo XXI se han desarrollado varios aspectos que inciden en la mejora de los EQA:

1) Naturaleza del material control: conmutabilidad y asignación de valores.
2) Tipos de EQA según su capacidad de evaluación.
3) Especificaciones para evaluar a los participantes.
4) Estandarización entre EQA.
5) Vigilancia del mercado.

A continuación, se comenta cada uno de esos aspectos.

#### Naturaleza del material control: conmutabilidad y asignación de valores

Un control conmutable es aquel que tiene la misma matriz que las muestras de los pacientes, por lo que se comporta igual que ellas frente a los diversos métodos analíticos existentes para determinar una misma magnitud biológica.

Como ejemplo, el programa holandés SKML desarrolló en el año 2000 un programa externo para 6 enzimas con muestra control conmutable, que distribuyó entre sus participantes junto con el programa regular (control no conmutable). Demostró que la dispersión entre laboratorios era inferior en el primer caso (CV entre 2.2% y 4.9%) que en el segundo (CV entre 4.6% y 10.8%) [22]. La SEQC estableció un convenio para distribuir su material control conmutable de bioquímica básica una vez al año desde 2015.

Ceriotti demostró en 2014 que, si se distribuyen controles conmutables y con valores asignados por métodos de referencia, se puede verificar la incertidumbre de las determinaciones y definir acciones correctivas adecuadas [23].

La forma segura de que un EQA pueda verificar la veracidad de los resultados es distribuir muestras humanas congeladas (sin ninguna otra manipulación) y conmutables, cuyos valores hayan sido asignados por los laboratorios de referencia listados en el *Joint Committee for Traceability in Laboratory Medicine* (JCTLM) (https://www.jctlm.org/), usando métodos de referencia cuando existen [24].

Asignar valores con métodos de referencia a controles no conmutables implica un uso inadecuado de los recursos, porque los métodos de rutina pueden comportarse de distinta forma frente a estos materiales que las muestras de pacientes, por lo que la información que producen no se puede trasladar a los resultados de los pacientes.

#### Tipos de EQA según su capacidad de evaluación

La norma europea EN 14136 publicada en 2004 no impone un único patrón de organización de EQA en Europa, pero sí insiste en que los EQA proporcionen datos que sean útiles para monitorizar las prestaciones analíticas de procedimientos específicos como, por ejemplo, identificar inequívocamente los procedimientos analíticos individuales y, sobre todo, distinguir entre características de las prestaciones propias de un procedimiento particular y aquellas atribuibles a sus usuarios [25].

El *Clinical and Laboratory Standards Institute* (CLSI) de USA, en el año 2008, perfiló las características de los EQA e incidió en su papel educacional, por ejemplo, informando al laboratorio sobre la posible repercusión para el paciente de sus resultados incorrectos [26].

En el año 2011 Miller y cols. identificaron los factores clave para evaluar la capacidad de los EQA: conocer la conmutabilidad de los controles distribuidos y saber el procedimiento utilizado para asignar sus valores diana. Establecieron seis categorías de EQA en función de su capacidad de evaluación [27]:

– Categorías 1 y 2: usan controles conmutables con valores asignados por métodos de referencia certificados. La primera analiza replicados de las muestras control, por lo que verifica tanto la veracidad como también la reproducibilidad del laboratorio, del grupo par (usuarios del mismo método, instrumento y reactivo) y del método, además de la trazabilidad a patrones de referencia dentro de la cadena de trazabilidad. Asegura la estandarización entre laboratorios. La segunda verifica la veracidad del laboratorio y del método, pero no la reproducibilidad.
– Categorías 3 y 4: usan controles conmutables, pero no con valores asignados por métodos de referencia. La primera con replicados de controles, por lo que evalúa la reproducibilidad del laboratorio y del grupo par, pero la segunda con análisis únicos de las muestras control, por lo que solo valora la reproducibilidad entre grupos. Ambas comparan la desviación existente entre los grupos par o entre métodos, pero no permiten evaluar la veracidad de las medidas ni la trazabilidad a patrones de referencia.
– Categorías 5 y 6: usan controles no conmutables y sin valores asignados por métodos de referencia. La categoría 5 analiza replicados de controles, por lo que verifican la reproducibilidad del laboratorio y la del grupo; la categoría 6 solo verifica la reproducibilidad del laboratorio.

Varios proveedores de EQA tienen programas donde aducen que sus controles son conmutables; sin embargo, un buen número utiliza programas de categorías 5 y 6. Además, no existen materiales y métodos de referencia para todos los mensurandos analizados habitualmente en el laboratorio médico.

#### Especificaciones de la prestación para evaluar a los participantes

Como se ha mencionado anteriormente, ya en 1996 el grupo europeo BCR había visto que un 56% de los programas EQA usaban el estado del arte, un 25% las opiniones de expertos (diversas y dispares) y un 19% la variación biológica [15]. Veinte años después, en 2017, una encuesta realizada por Jones y cols. destacó que la gran mayoría usaban la variación biológica (42%) y el estado del arte (38%) [28].

El programa australiano para hormonas utilizó en 2004 las especificaciones derivadas de la variación biológica para evaluar la imprecisión, el sesgo (error sistemático) y el error total analítico de sus participantes. Concluyó que el uso de límites fijos en EQA facilita la identificación de la fuente de error en el laboratorio participante, clarifica la evaluación de métodos analíticos y unifica la información obtenida por distintos EQA. Este trabajo señala como limitación el uso de controles no conmutables, puesto que pueden dar una información no transferible al espécimen humano [29].

A raíz de una reunión de expertos de Europa, Israel y Sudáfrica en 2011 en la que se debatieron las especificaciones a usar en EQA, se concluyó que las derivadas del estado del arte son adecuadas para prestaciones extra-analíticas, mientras que las derivadas de la variación biológica son recomendables para la prestación analítica; así mismo los laboratorios deberían presionar a la industria de diagnóstico *in vitro* para que sus sistemas analíticos alcancen estas especificaciones [30].

En 2015 Jones señaló la falta de armonización de las especificaciones de prestación analítica en los EQA, aún quince años después del consenso internacional de Estocolmo. Insistió en que los EQA deberían informar a sus participantes acerca de la naturaleza de las especificaciones con que los evalúan, así como del motivo de dicha elección [31].

En la conferencia estratégica EFLM (Milán 2014) se decidió caminar hacia un objetivo para los EQA: armonizar las especificaciones para evaluar a los participantes, y así expandir los mismos mensajes sobre la calidad de la prestación, independientemente del laboratorio y del EQA elegido.

El grupo de trabajo de expertos *EFLM Task Finish Group – Analytical Performance Specifications for EQA* recomendó que todos los EQA informasen a sus participantes sobre 6 aspectos [32]:

– Matriz del material control distribuido y su conmutabilidad.
– Método utilizado para asignar valor diana al control.
– Datos a los que se aplican las especificaciones (desviación del laboratorio individual, desviación del grupo de laboratorios con el mismo método, etc.).
– Variable analítica que se evalúa (imprecisión, error sistemático, error total).
– Tipo de especificación (variación biológica, estado del arte, etc.).
– Motivo por el que se ha elegido la especificación.

Existen EQA para pruebas en escala nominal u ordinal, en los que los laboratorios se evalúan sobre la base del porcentaje de respuestas correctas obtenidas durante un ciclo. Si las pruebas en escala ordinal se transforman en cuantitativas, las especificaciones para evaluar resultados son las descritas anteriormente [33].

#### Estandarización entre EQA

Los cinco pilares que sostienen la estandarización entre laboratorios médicos se identificaron como [34], [35], [36]:

– Materiales de referencia certificados.
– Procedimientos de medida de referencia.
– Laboratorios de referencia reconocidos.
– Intervalos de referencia consensuados.
– Programas EQA comparables.

Acerca del quinto pilar que concierne a los programas EQA, los autores insistieron hace ya una década que, para conseguir la estandarización de las medidas en los laboratorios médicos, los EQA deberían ser comparables entre sí.

A ese respecto, la capacidad de los EQA categoría 1 para evaluar la estandarización de las prestaciones fue investigada por Jansen y cols. en un estudio con laboratorios de Holanda, España, Portugal y Reino unido. Evaluaron el error total analítico (con especificaciones de cumplimiento basadas en la variación biológica) y pusieron de manifiesto importantes discrepancias para 11 de las 18 magnitudes biológicas estudiadas (calcio, cloruro, magnesio, sodio, ALT, amilasa, AST, LDH, colesterol HDL, creatinina y proteínas totales). Vieron que para muchas de ellas había discrepancias relacionadas con los instrumentos o plataformas analíticas empleadas y, en algunas, también incluso entre usuarios de un mismo instrumento [37, 38].

Así mismo, los estudios de De Grande y cols (Empower project) [39] comparando varios grupos de muestras congeladas de un único donante y monitorizando los percentiles de los resultados de pacientes emitidos por varios laboratorios, aportan una visión realista de la inter-comparabilidad entre ensayos.

Ricós y cols [28]. evaluaron los resultados durante 4 ciclos de distribución en España de un EQA de categoría 1 y vieron que solo 2 de los 18 mensurandos estudiados se podían considerar bien estandarizados (CK, potasio) ya que el sesgo existente entre grupos pares cumplía la especificación para error sistemático derivada de la variación biológica.

La falta de estandarización para 8 de los mensurandos era debida a diversos motivos:

– ALP y proteínas incluso utilizando todos los laboratorios el mismo método, mostraban diferencias entre instrumentos.
– ALT, AST, amilasa, creatinina, GGT y LDH evidenciaban desviaciones inadmisibles para algunos métodos concretos: ALT y AST en aquellos formulados sin piridoxal fosfato, para la medición de amilasa los métodos con sustrato de malto triosa, la medición de creatinina con el método de Jaffé, GGT con los métodos que utilizaban el sustrato <4 mmol, y la medición de LDH con el método reverso (piruvato-lactato).

En el 2021 (datos no publicados), se estudiaron los 8 mensurandos no estandarizados sin causa aparente (ciclos 2015–2019), enfocando únicamente la concentración de interés clínico. Se observó que:

– Magnesio y urato estaban bien estandarizados a esta concentración. Urato se determinó con un único método analítico (uricasa-POD), pero magnesio lo hizo con tres métodos (azul xilidilo, azul metil timol y enzimático), indicando que la variedad de métodos analíticos no es necesariamente un factor limitante para la estandarización. Ambos mensurandos eran trazables a métodos de referencia (IDMS en urato, Absorción atómica en magnesio), mostrando como la trazabilidad a método (que obvia el posible efecto matriz de un material), favorece la estandarización.
– Bilirrubina, calcio y glucosa estaban bien estandarizados solo en algunos ciclos. La no continuidad de la estandarización podría deberse a cambios de lote del calibrador de rutina o de los materiales intermedios del sistema de medida de referencia [40], así como a la laxa incertidumbre admitida por el fabricante para sus materiales intermedios, como apuntan Braga y cols [41].
– Para cloruro y sodio medidos por ISE, la estandarización es factible en cloruro, pero nula en sodio.

#### Vigilancia de los métodos analíticos disponibles en el mercado

El papel de vigilancia otorgado a los EQA por la Directiva Europea 98/79/EC fue aplicado por el programa belga, que evidenció, en un seguimiento de 10 años con control estabilizado las siguientes “sorpresas” [42]:

– Valores discriminantes y valores de referencia biológicos descritos en los “*insert*” de los fabricantes, inadecuados.
– Reacciones no específicas en algunos lotes de reactivos.
– Corrosión causante de interferencias en algunos dispensadores de algunos analizadores automáticos.
– Equipos (*kits*) de reactivos con prestación inadecuada.

**Figura 1:**
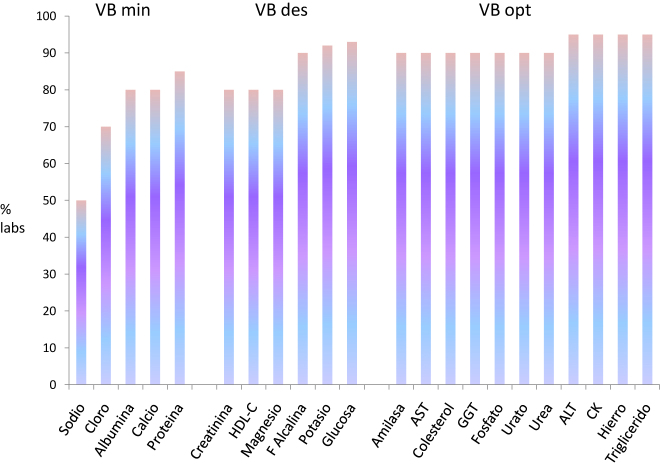
Cumplimiento de las especificaciones para el error total derivadas de la variación biológica en el programa Bioquímica-suero. Datas obtenidos de Perich et al. [43].

**Figura 2:**
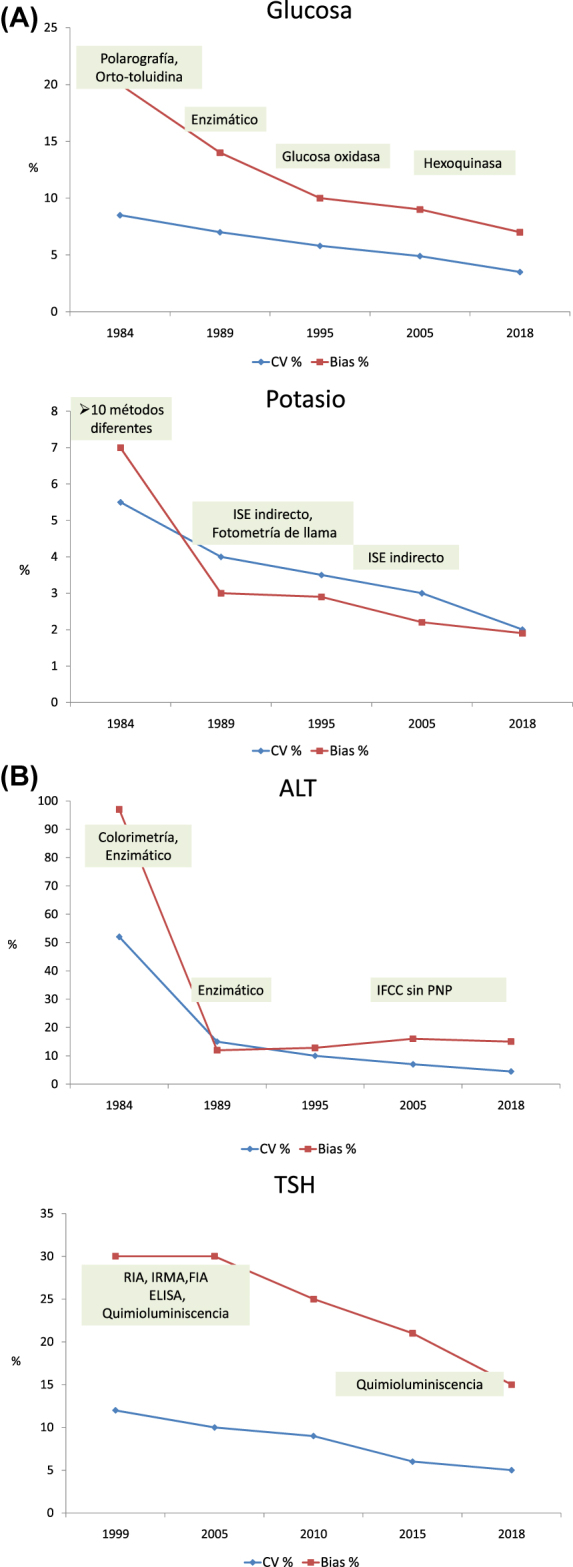
Evolución del CV (%) y el sesgo (%) con el cambio de metodología. (A) Glucosa y potasio. (B) ALT y TSH. Datos obtenidos de Perich et al. [43].

Perich y cols [43]. estudiaron la evolución de los resultados de los programas EQA de la SEQC con suero control liofilizado durante más de 30 años (1981–2018). Observaron una mejora de las prestaciones de los laboratorios participantes, con una disminución de las desviaciones con el paso del tiempo, p.e. en el programa de bioquímica del año 2018 el 90% de los participantes alcanzaron las especificaciones derivadas de la variación biológica e incluso el 50% o más alcanzan las de los mensurando con más fuerte regulación homeostática (Figura 1). También evidenciaron disminución de la imprecisión y el sesgo entre laboratorios debida a los cambios en la metodología analítica usada por los participantes (Figuras 2A, B). Como nota negativa, pusieron de manifiesto la persistencia de métodos obsoletos o poco específicos cuando hay disponibles otros métodos con prestaciones demostradamente superiores (p.e. creatinina con Jaffé o transaminasas por método IFCC, pero sin piridoxal fosfato).

La imprecisión inter-laboratorios fue similar a la obtenida en los EQA holandés (Dutch Stichting Kwaliteitsbewaking Medische Laboratoriumdiagnostiek (SKML) [44], belga (Empower Project) [45] y alemán (German Referenzinstitut für Bioanalytik) [46]. Las dificultades para poder comparar resultados entre EQA fueron:

– Solo se pudo obtener información de programas que los publican en revistas indexadas o en páginas web accesibles a no participantes.
– Uso de distintos criterios para formar grupos par.
– Realización de cálculos estadísticos diferentes.
– Aplicación de especificaciones diversas para evaluar los resultados.

### Fortalezas y debilidades de los EQA

Ceriotti y Cobbaert se plantearon en 2018 tres cuestiones relativas a los EQA [47]:

– ¿Cumplen lo que se espera de ellos?
– ¿Todos los EQA son equivalentes, es decir están armonizados?
– ¿Debe el papel de los EQA ser igual para todas las disciplinas del laboratorio médico?

La respuesta a la primera pregunta es un no, porque muchos EQA no verifican la veracidad de la prestación del laboratorio al no usar controles conmutables con valores asignados por métodos de referencia. Solo constatan la comparabilidad de las prestaciones entre grupos par.

La respuesta a la segunda pregunta también es un no: la armonización entre EQA no existe debido, entre otros aspectos, a distintos tipos de materiales control usados y, sobre todo, a distintos criterios de evaluación. Esto es debido a los distintos modelos de EQA, desde obligatorios con sanciones para los participantes con evaluación insuficiente, hasta educativos que empujan a perfeccionar la prestación. La situación podría mejorar si se implementara la acreditación de los EQA por la norma ISO 17043:2010 [48].

La respuesta a la tercera pregunta es un sí: es necesaria información sobre la veracidad y reproductibilidad de todas las determinaciones realizadas en el laboratorio médico.

Sciacovelli y cols estudian los aspectos clave que los EQA deberían cumplir para facilitar a los laboratorios el acceso a la acreditación por la norma ISO 15189 [49]:

– Definir las mejores especificaciones de la prestación posibles para cada mensurando y que estas fuera adoptadas en todos los EQA.
– Ayudar a los laboratorios para encontrar alternativas a un EQA, cuando no hubiera ninguno disponible.
– Describir claramente en su informe las causas de prestación inadecuada, especialmente si fueran debidas al propio laboratorio o al procedimiento analítico utilizado.

Badrick y Stavelin [50] constataron que al coexistir los dos modelos generales de EQA (obligatorios por ley y educativos), un laboratorio que participe en ambos tipos de programas puede conseguir prestación aceptable en uno de ellos, pero inaceptable en el otro, para un mismo mensurando. Los autores abogan por la creación de una entidad que lidere una armonización entre EQA y sugieren la ya existente *European Organisation for External Quality Assurance Providers in Laboratory Medicine* (EQALM) (http://www.eqalm.org), que tiene la confianza de muchos organizadores de EQA.

Van der Hagen y cols [51]. estudian la capacidad de agregar (combinar) datos procedentes de EQA de 4 países (USA, Noruega, Holanda, Reino Unido) con controles conmutables y valores asignados por método de referencia para creatinina. Constatan que es posible agregar los datos, siempre y cuando la descripción de los métodos y sistemas analíticos sea la misma en todos los EQA. Insisten en que en un futuro, habría que describir la conmutabilidad de los controles de una forma común y estandarizar mejor la información recibida de los participantes (p.e calibración, lotes de reactivos, etc.) para optimizar la clasificación de la prestación de los distintos sistemas de medida del mercado. Recientemente, el *International Consortium for Harmonization in Laboratory Medicine* (ICHCLR) y la EQALM, han puesto en marcha una iniciativa conjunta denominada HALMA para verificar la armonización de los mensurandos mediante la agregación de datos procedentes de diversos programas EQA a nivel internacional (http://www.eqalm.org/site/halma/halma.php).

Las ventajas e inconvenientes de los programas EQA más usados han sido manifestados por Jones y cols [52] (Jones CCLM 2022) y se resumen en la Tabla 1, presentada por González-Tarancón y cols. en una sesión de la Academia-SEQC [53].

**Tabla 1:** Ventajas e inconvenientes de los programas EQA más usados.

Tipo de EQA	Ventajas	Inconvenientes
1 y 2	– Conocer imprecisión y sesgo real y poder extrapolar a resultados de pacientes.	– Caro
	– Verificar estandarización de los métodos y comprobar si los resultados son intercambiables.	– Requisitos de conservación
	– Identificar laboratorios y métodos con prestaciones deficientes.	
	– Erradicar métodos no estandarizados, promover el uso de métodos estandarizados, o en su defecto armonizados, y demostrar que su uso mejora la seguridad del paciente.	
	– Verificar la validez de los datos de pacientestomados rutinariamente para su uso posterior en estudios por métodos indirectos (big data).	
5 y 6	– Comparación entre iguales	– No exactitud
	– Armonización	– No estandarización
	– Barato	
	– Monitorización laboratorio individual	
	– Mismo lote control en el tiempo	
	– Monitorización de un fabricante	

Obtenido de González-Tarancón R y cols [53].

### Recomendaciones para los participantes en EQA

El CLSI da recomendaciones a los participantes para usar adecuadamente los programas EQA [26]. Así, entre otras:

– Revisar cada informe EQA y observar la desviación de su laboratorio respecto a la media del grupo par y elaborar gráficos de seguimiento de los diversos resultados en un mismo ciclo.
– Observar la dispersión de los distintos grupos par, para conocer cuál es el más preciso.
– Tratar los errores cometidos, para discriminar si son de tipo administrativo, del procedimiento analítico, del método usado, de las habilidades del personal o inexplicable.
– Implantar acciones correctivas y evaluar su efecto en el informe EQA.

Si el laboratorio participa en un programa de categoría 1 puede hacer varias cosas más:

– Conocer exactamente el efecto del impacto que cada desviación (calibración, variación lote a lote, etc.) tiene sobre sus pacientes.
– Verificar su inexactitud estudiando su desviación con respecto al valor de referencia del control.
– Poner de manifiesto el método analítico disponible más exacto.

Cuando no existe EQA para una determinada magnitud biológica, se pueden intercambiar muestras de pacientes con otros laboratorios e interpretar los resultados siguiendo las recomendaciones del CLSI-EP-31-A-IR [54].

### Como debe el laboratorio elegir un EQA

Miller y Sandberg [33] sugieren que el laboratorio se plantee las siguientes seis preguntas:

– ¿Cuánto se parece el material control distribuido por el EQA a la muestra de paciente?
– ¿Es un material control conmutable?
– ¿Cuántos replicados controles se miden en cada evento?
– Cómo se ha establecido el valor diana del material control?
– ¿Cuantos participantes hay en cada grupo par?
– ¿Cómo se han establecido las especificaciones de la prestación?

Siempre que sea posible, habría que participar en programas EQA con controles conmutables y valores asignados por métodos de referencia certificados; éstos existen para unos 110 mensurados [55]. De este modo el laboratorio conoce la veracidad de sus prestaciones y el EQA constata qué métodos están estandarizados. Esto es de vital importancia para los mensurados que se interpretan sobre la base de un valor de decisión clínico.

Si se participa en EQA con controles no conmutables, el laboratorio se compara con su grupo par; esto permite al laboratorio saber si puede compartir valores de referencia poblacionales. Sin embargo, aún dentro de este grupo pueden existir discrepancias entre distintos lotes de reactivos, aunque las muestras de pacientes den resultados similares.

Si el EQA contempla análisis replicados del material control distribuido, el laboratorio tendrá información sobre su reproducibilidad.

La incertidumbre del valor diana es mayor en EQA con controles no conmutables, porque depende del número de laboratorios que forman el grupo par y la imprecisión de los métodos de rutina es mayor que la de los métodos de referencia.

### EQA en mediciones a la cabecera del paciente (point-of-care testin, POCT)

Es muy importante que las determinaciones POCT estén sometidas a las mismas estrategias de aseguramiento de la calidad que el laboratorio central (control de calidad interno y EQA), pues con estas mediciones se toman decisiones clínicas y el laboratorio es el indicado para gestionar esta información.

Si embargo, en ocasiones las prestaciones alcanzadas por los equipos POCT no son las mismas que las de los métodos utilizados comúnmente en el laboratorio central (caso de la HbA_1c_) y además no están dirigidas a un mismo uso clínico, por lo que estos equipos habrían de constituir un grupo específico en sí mismo y las especificaciones de cumplimiento pudieran ser diferentes.

Es más, lo ideal es que existan EQA diseñados específicamente para redes POCT. NOKLUS tiene mucha experiencia y el compromiso y los resultados logrados tras la formación impartida han sido espectaculares. Otros proveedores como INSTAND (Alemania), WEQAS en cooximetría y bilirrubina (Gales) o Labquality para glucosa capilar (Finlandia) también tienen este tipo de programas específicos.

### Qué debe hacer el laboratorio

Si el laboratorio es responsable de laboratorios satélite con sistemas analíticos ajustados entre sí mediante factores de corrección, debe enviar al EQA los valores habiendo anulado previamente el factor de corrección; de otro modo, no existirá un grupo par con el cual pueda comparar sus resultados.

En ningún caso el laboratorio debe ajustar su procedimiento analítico al material control de EQA, porque pierde la trazabilidad del calibrador que es responsabilidad del fabricante.

## Conclusiones

En resumen, el laboratorio debe ser consciente de:

– Si quiere conocer la inexactitud real de sus resultados y el impacto en las muestras de pacientes, así como la trazabilidad de sus calibraciones, debe participar en un EQA con controles conmutables y valores asignados por método de referencia certificado (EQA categoría 1).
– Si solo quiere conocer si su prestación es similar a la de otros laboratorios con su mismo método analítico, debe participar en programas con controles no conmutables; sin embargo, éstos deberían ser conmutables entre distintos lotes de reactivo.

Los EQA deben consolidar:

– Ofrecer programas con controles conmutables y valores asignados por métodos de referencia certificados, que verifican la estandarización entre laboratorios.
– Informar a sus participantes que si distribuyen controles no conmutables sólo evalúan la armonización entre laboratorios, pero no su estandarización.

En este caso, el criterio de aceptabilidad debería ampliarse teniendo en cuenta la incertidumbre del valor diana.

– Nominar una entidad que promueva la armonización entre programas EQA.
– Agregar los resultados de distintos organizadores de EQA para promover la armonización entre EQA a gran escala.
– Pedir a los participantes el registro de lotes de reactivos y de calibradores, porque los métodos de rutina pueden comportarse de forma distinta frente a lotes diversos.
